# Online Recruitment Methods for Web-Based and Mobile Health Studies: A Review of the Literature

**DOI:** 10.2196/jmir.4359

**Published:** 2015-07-22

**Authors:** Taylor S Lane, Julie Armin, Judith S Gordon

**Affiliations:** ^1^ University of Arizona Department of Family and Community Medicine Tucson, AZ United States

**Keywords:** mHealth, Internet health, online recruitment, apps, social media, review

## Abstract

**Background:**

Internet and mobile health (mHealth) apps hold promise for expanding the reach of evidence-based health interventions. Research in this area is rapidly expanding. However, these studies may experience problems with recruitment and retention. Web-based and mHealth studies are in need of a wide-reaching and low-cost method of recruitment that will also effectively retain participants for the duration of the study. Online recruitment may be a low-cost and wide-reaching tool in comparison to traditional recruitment methods, although empirical evidence is limited.

**Objective:**

This study aims to review the literature on online recruitment for, and retention in, mHealth studies.

**Methods:**

We conducted a review of the literature of studies examining online recruitment methods as a viable means of obtaining mHealth research participants. The data sources used were PubMed, CINAHL, EbscoHost, PyscINFO, and MEDLINE. Studies reporting at least one method of online recruitment were included. A narrative approach enabled the authors to discuss the variability in recruitment results, as well as in recruitment duration and study design.

**Results:**

From 550 initial publications, 12 studies were included in this review. The studies reported multiple uses and outcomes for online recruitment methods. Web-based recruitment was the only type of recruitment used in 67% (8/12) of the studies. Online recruitment was used for studies with a variety of health domains: smoking cessation (58%; 7/12) and mental health (17%; 2/12) being the most common. Recruitment duration lasted under a year in 67% (8/12) of the studies, with an average of 5 months spent on recruiting. In those studies that spent over a year (33%; 4/12), an average of 17 months was spent on recruiting. A little less than half (42%; 5/12) of the studies found Facebook ads or newsfeed posts to be an effective method of recruitment, a quarter (25%; 3/12) of the studies found Google ads to be the most effective way to reach participants, and one study showed better outcomes with traditional (eg in-person) methods of recruitment. Only one study recorded retention rates in their results, and half (50%; 6/12) of the studies recorded survey completion rates.

**Conclusions:**

Although online methods of recruitment may be promising in experimental research, more empirical evidence is needed to make specific recommendations. Several barriers to using online recruitment were identified, including participant retention. These unique challenges of virtual interventions can affect the generalizability and validity of findings from Web-based and mHealth studies. There is a need for additional research to evaluate the effectiveness of online recruitment methods and participant retention in experimental mHealth studies.

## Introduction

### Background

Chronic health conditions have replaced acute diseases as the leading causes of both illness and death in the United States [1]. While overall mortality rates have declined, chronic health conditions are becoming more common [1]. There is a great need for empirically based treatment and support for these conditions [2]. While the accessibility and availability of health information is limited when it is delivered solely in medical settings, the Internet and mobile health (mHealth) apps hold promise for expanding the reach of evidence-based health interventions and information [3]. Both tools have opened up a new audience for health information [4]. According to a recent survey, approximately 284.5 million people in the United States (90% of the population) have at least one mobile phone [5]. An estimated 58% of mobile phone users have a smartphone, about 31% of mobile phone owners use their phones to look for health or medical information, and approximately 35% of mobile phone users download apps to track or manage their health [6,7]. A similar report found that 72% of adults look on the Internet for health information [8]. Sixty-five percent of Internet users say they are better informed about health because of Internet and mobile phone use, and 44% report that these technologies have greatly helped their ability to get this information [9]. mHealth apps hold power in their accessibility and reach [10], as well as their ability to lower the costs associated with relaying information, support, and assistance to those who need it [11]. While in-person interventions and consultations produce the highest efficacy, mobile technologies may address the disparity between supply and demand for health-related services and information, thus creating a potentially lower-cost solution to bridging that gap [4,12]. In addition, mHealth apps and Web-based interventions provide flexibility that in-person interventions are unable to provide, such as around-the-clock access to information and personalized feedback [4,13,14]. The benefits of this technology are promising but are in the development stage. However, mHealth is predicted to continue its growth as the technology becomes more pervasive in the US population [3].

Although research has been conducted for many years on Web-based health interventions, research in the area of mHealth is nascent. There are few published studies of mHealth apps designed using theoretical frameworks [4,14-16]. There are a large number of smoking cessation apps available for Apple, Android, and Windows Phone platforms, but the vast majority do not include basic evidence-based practices and are found to have low levels of adherence, meaning that participants quit using the app over time [17,18]. In addition, a majority of Web-based smoking cessation interventions are not evidence-based [19]. There is a clear need for evaluation of Web-based and mHealth interventions to ensure that public health problems, such as smoking, can be addressed with evidence-based tools [17,19].

Web-based interventions and mHealth apps are fundamentally different, but research in these areas can experience similar challenges. There are special issues involved with conducting virtual interventions. Research on mHealth apps and Web-based interventions is difficult due to unique challenges associated with recruiting, enrolling, and retaining participants [4,13]. Recruitment and retention of participants in these studies directly affects sample size, which determines the power and significance of the study [14,20]. If recruitment goals are not met in the initial period, it can negatively influence the chance of finding an effect or, if an extension on the recruitment period is required, it can expand study costs considerably [20]. Participant retention is another main concern for studies using mHealth interventions [4]. Some claim attrition may be due to early interest in the novelty of mHealth apps, which then fades as the innovation wears off [4], while others suggest the lack of personal contact can lead to higher dropout rates [21]. Attrition is inevitable, especially in lifestyle interventions such as diet or smoking cessation where dropout rates less than 20% are rarely achieved, but excessive attrition can reduce study power, increase bias, and lower generalizability [22].

Challenges in enrolling participants have led researchers to look towards alternative methods for identifying potential participants [23,24]. Web-based strategies for attracting participants, such as the use of Internet advertising, email invites, craigslist, online message boards, and more recently, social media, have been explored by researchers in order to find general and specific populations for studies [24]. Some studies indicate that online strategies can be effective at reaching a larger number of potential participants, as well as improving affiliated costs [4,13,24]. Online recruitment can cast a broader net than traditional recruitment, extending to previously hard-to-reach populations, such as young adults, racial/ethnic minorities, and those with low education attainment [25,26]. In addition, Internet-based recruitment can be affordable and reach a wider, more diverse population, thus increasing generalizability [27,28]. However, there is lack of consistency in reporting these population variables in studies employing these methods [29].

In a recently completed study (National Cancer Institute Grant #R41CA162502; J Gordon, PI), our research team developed and evaluated a theory-based mHealth app to improve medication adherence and provide behavioral support for tobacco cessation. Our team used online and in-app methods of recruitment with varying degrees of success. The research team, currently conducting another study (National Cancer Institute Grant #R21CA174639; J Gordon, PI) to test a multi-behavioral smoking, diet, and physical activity mHealth intervention, sought to identify the most effective methods for recruiting and retaining research participants. Our first-hand experience, as well as the desire to formulate recommendations for other researchers, prompted an analysis of the literature on methods of attracting and retaining participants in mHealth studies.

### Variables of Interest

The cost of the recruitment method used and participant retention rates were our primary variables of interest. Recruitment cost can be important with a limited budget in order to find the largest, most representative recruitment sample with the allotted funds. Participant retention rates were a primary focus due to our suspicion that the lesser amount of investment required from participants in virtual research negatively affects retention [4,21]. In addition, the broad reach achieved by Web-based recruitment might bring in people who are unfamiliar with the research environment and are unaware of the extent of the commitment they are making. This might later affect the number of participants who enroll and complete all interventions. This predicted higher attrition rate was evident in our own experience, as well as in the literature on Web-based and mHealth studies [4,21]. Participant demographics were included in the review to allow for comparison to “traditional” methods of recruitment. The “traditional” recruitment methods are defined as telephone, newspapers, radio, TV, flyers, or word-of-mouth. Other variables included were duration of recruitment and intervention because of their influence on participant retention and engagement, which are important factors in the generalizability and success of mHealth research [4,22].

## Methods

### Search Strategy

A review of the literature published between 2004 and 2014 was conducted from August 2014 to October 2014, using the search terms “mHealth”, AND “social media”, AND “health behavior change”, AND “online recruitment”, OR “recruitment”, in the databases PubMed, MEDLINE, Cumulative Index of Nursing and Allied Health Literature (CINAHL), PsycINFO, and EbscoHost. Articles’ reference lists were also screened for relevant literature. Two searches of the literature were conducted, with the first using more stringent selection criteria. The goal of the initial search was to focus solely on mHealth research. The second search was conducted with more expansive selection criteria to include Web-based interventions due to minimal results from the first search.

### Phase I Selection Criteria

The initial review was limited to articles that were published in English, had an abstract available, and reported on research about behavioral interventions to improve health. While certain demographic components such as age can affect recruitment results [23], there were no specifications for age or race of participants included in the review. The first author (TL) screened the abstracts and titles of relevant articles for eligibility. Only peer-reviewed papers were included. Mobile health articles that detailed their recruitment process in their methods section were selected (TL). These articles reported online recruitment methods and outcomes from current or past experimental studies, retention rates, and costs related to online recruitment. This initial search produced only one publication [29].

### Phase II Selection Criteria

The first search highlighted the novelty of the mHealth research field. Therefore, an additional, broader search was conducted to see if any Web-based studies, in addition to mHealth studies, published articles detailing their recruitment processes and outcomes. This search utilized the same keywords and similar study selection criteria. The only change was expanding the criteria to include Web-based studies, as well. This second phase search gave the researchers a better understanding of what types of articles were being published about online recruitment and what types of information were being reported. Upon conclusion of the broader search, 11 peer-reviewed publications were included for the literature review. This created a total of 12 peer-reviewed articles [24-27,29-36].

### Descriptive Characteristics of the Studies

The following data were collected from each article and recorded in an Excel spreadsheet: (1) authors of the publication, (2) year of publication, (3) article digital object identifier (DOI) number, (4) health domain, (5) study design, (6) number of participants enrolled in the study, (7) general demographics of participants, (8) method of recruitment, (9) total time duration of recruitment, (10) total cost of advertising, as well as cost per click if applicable, (11) percentage retention of participants, (12) study or intervention duration, and (13) most effective methods of recruiting, including cost and number of participants, found by the study.

## Results

### Description of Studies

#### Overview

Four of the articles (33%) were published in 2014, three (25%) were published in 2013, and the rest were published between 2006 and 2012. The most common health category was tobacco use/smoking cessation (58%, 7/12). Other categories included mental health, general health, and human immunodeficiency virus (HIV). One third (4/12) of the studies used a randomized control trial as their study design, and one quarter (3/12) used a comparison study design. Other designs included exploratory design, pilot studies, and feasibility trials. The most common participant age group recruited was 18-25 years old. One study included participants between 16-25 [25], and all other research employed broad criteria, requiring participants to be older than 18 years of age. The studies targeting users under 25 used Facebook advertising only, whereas all other studies used a variety of online and traditional methods. Examples of online recruiting methods consist of paid media, including search engine advertising, and earned media, such as posting on various websites and craigslist. A majority (83%, 10/12) of the studies recorded participant engagement in some way. An example of engagement could be the number of page or icon clicks within an app in an mHealth study (see the Participant Engagement and Retention section for a more in-depth explanation).

#### Recruitment Method

Five of the articles used only one recruitment tool (42%), which was Facebook. These articles specified that they used Facebook, as they were targeting only a small demographic (eg, individuals 18-25). Three of these articles [30-32] used different types of ads through Facebook (17%), some more successful than others. The first article, which looked at depression, found that ads that were closely aligned with the content of the research and used wording regarding a problem to solve were more successful than the other ads [30]. The second article, which looked at smoking cessation, found that newsfeed ads were more successful. They also found that simple images of cigarettes, the study logo, and general informational messaging were the most successful compared to more complex images and intricate targeted messaging [31]. The third article, which looked at tobacco use, found that Facebook ads were more affordable than previous methods used. They noted success of ads as being dependent upon Facebook’s approval of the ad [32]. A quarter (3/12) of the articles used multiple methods of Web-based recruitment [27,33,34]. One study advertised through search term query results on multiple search engines [34], and the other two studies used a combination of various websites, search engines, and social media outlets for recruitment purposes [27,33]. One third of the studies (4/12) used a combination of traditional methods and Web-based methods of recruitment and compared them side-by-side for effectiveness [24,26,29,35]. Two studies [24,35] used Facebook ads as the Web-based method (17%) and compared it to the use of flyers and newspaper ads. One study [26] used national websites, local websites, and search engines, and compared them to the use of billboards, TV and radio ads, and direct mail. One study [29] used health websites (eg, WebMD.com), Google AdWords, Facebook, and Twitter, and compared them to TV, radio, newspaper, email, and word of mouth.

#### Recruitment Duration and Participant Numbers

Recruitment duration was deemed to be an important factor due to its direct impact on the costs associated with recruitment methods. The majority of articles (67%, 8/12) reported an average recruitment duration of approximately 5 months (range 7 weeks-7 months). Of the studies that recruited for less than a year, half noted two trial periods of recruitment where advertising was changed in some way after the first trial. Two studies (17%) noted the reason for the split in recruitment period as a way to evaluate the effectiveness of different types of ads [30,31]. Articles that specified recruitment duration of less than 1 year had an average of 468 participants. Four articles (33%) reported an average duration of 17 months (range 13-23 months). Studies that recruited for more than 1 year had an average of 3199 participants.

#### Participant Eligibility

Eligibility criteria were diverse across articles, except for age (>18 years old), which was common across studies (92%, 11/12). Only one study recruited participants under age 18 [25]. Eligibility criteria were specific to the health domain on which the study focused. A majority of the tobacco cessation studies specified eligibility criteria (86%, 6/7), including English literacy, current resident of the United States, some form of Internet access, current smoking (usually defined by number of cigarettes smoked within a specific time frame), and not having used a specific tobacco cessation website or other intervention. Half (6/12) of all articles specified geographic location as an eligibility requirement, usually the country in which the study was being conducted. Half (6/12) of all articles also had intervention-related criteria as eligibility requirements. Examples of intervention-related criteria include access to the Internet for some period of time in a week or not already receiving treatment for the health problem specified in the study. One article did not include any eligibility requirements [26].

#### Recruitment Costs

Cost was recorded in several ways: as overall recruiting cost, cost-per-click for paid advertising on social media sites and search engines, cost per participant or per completed survey, and/or direct cost related to the specific type of recruitment tool used (eg, Facebook ads). A majority (80%, 4/5) of the studies that used Facebook ads as their sole method of recruitment reported that the advertising features on Facebook helped their recruitment (Table 1). These four studies used Facebook ads successfully to recruit their ideal number of subjects within their budget and reported the ability to target a specific population or demographic as an important factor [25,30-32]. One study that compared Internet advertisements to craigslist and email invitations found Facebook to be the most effective type of Internet ad as it recruited the most participants in the 18-25 year old age range [33]. However, one study that used Facebook as the sole method of recruiting found this strategy was not able to generate sufficient participation for conducting large sample surveys [36]. Two Web-based recruitment studies (17%) did not specify a target age range and used Google ads to successfully to recruit ideal participant numbers while staying within budget [27,34]. One compared advertising on Google to other search engines [34], and the other compared advertising on Google to social media outlets and online forums [27]. Certain search terms, such as those related to seeking a depression test or information about symptoms of depression [27], and “quit smoking” [34] were more effective in converting number of ad views to enrolled participants in both of these studies.

The studies that compared Web-based methods and traditional methods of recruitment reported various findings. One study recorded the highest yield of participants from Facebook, but found online advertising to cost twice as much per participant than using newspaper advertising, fliers, and word of mouth [24]. Another comparison study achieved the highest yield of participants from print advertisements, and found lower cost per participant from print ads and flyers than from Web-based methods [32]. However, this study noted that social networking sites were more likely to reach typically unrepresented populations [32]. The third comparison study reported that Google produced the highest yield of participants with the least cost, followed by news stories on medical Internet media (eg, websites such as WebMD) and traditional media [29]. This study also noted that using paid advertising and free posting on social media was the least cost effective [29].

**Table 1:** Summary of studies—Study type, participants, and recruitment.

Authors (year)	Study type	N	Participant eligibility	Method of recruitment	Recruitment duration	Recruitment costs
Batterham (2014)	Systematic Investigation	1893	Australian, ˃18 years old	Facebook advertising: First period—4 ads with direct link (2x2 factorial design- “problem” vs “positive” terminology and “altruistic” vs “self-gain” terminology). Second period- Facebook page that you “liked” and page showed visible links to the survey site. Facebook compared to previously completed study which used postal and telephone surveys.	First period recruited for 1 month. Second period recruited for 2 months.	Direct link cost $9.82 per completed survey. Survey linked to the Facebook page cost $1.51 per completed survey.
Fenner (2012)	Exploratory study	278	Female, 16-25, live in Victoria, Australia, willing to complete health survey	Facebook advertising	4 months	Cost-per-click: $0.67. Cost-per-participant: $20.14
Frandsen, Walters, & Ferguson (2013)	Multisite randomized trial	266	Older than 18, smoke more than 10 cigarettes a day for the last 3 years at least, not enrolled in another smoking cessation trial in the last 3 months, highly motivated to quit (>75 on 100 pt scale)	Two methods: Traditional—flyers, newspaper ads Online—Facebook ads	18 months	Average cost-per-click was AUD $0.95. Cost-per-participant was AUD $42.34. Newspaper cost-per-participant was AUD $21.52
Graham, Bock, Cobb, Niaura, & Abrams (2006)	Randomized controlled trial	764	Older than 18, smoking 5 or more cigarettes a day, no prior QuitNet.com use	Major search engines (AOL, MSN, Yahoo, Google), using an active user-intercept protocol	At least 7 months (recruitment was ongoing at published time)	[Not reported]
Graham, Milner, Saul, & Pfaff (2008)	Comparison of different media campaigns	9,655	Not reported	Two methods: Traditional—billboards, TV, radio, direct mail, physician detailing Online—national websites (banner ads), local websites (banner ads), paid search ads (per click basis)	One 6-month period, one 3-month period	First phase: $35 per participant (with a 9% conversion rate). Second phase: $38 per participant (with a 7% conversion rate)
Heffner, Wyszynski Comstock, Mercer, & Bricker (2013)	Pilot study	222	Older than 18 years, smoked at least five cigarettes daily for the past year, want to quit in the next 30 days, willing to be randomly assigned to either group, lives in the United States, has weekly access to the Internet, English literate, not participating in other smoking cessation interventions, and never used Smokefree.gov website	Two methods: Traditional—Standard media (news coverage on TV, radio, newspaper ads), emails, word of mouth. News coverage on online—medical Internet media, Google AdWords, Facebook advertising and free posts Twitter posts	10 weeks	Total cost: $9,429.83; Direct cost from Facebook: $1,250; Direct cost from Google: $3,320.53; Direct cost from press releases: $1,250; Cost-per-participant: $42.48.
Morgan, Jorm, & Mackinson (2013)	Randomized controlled trial	1326	˃18 years, from Australia, New Zealand, UK, Ireland, Canada, or US, access to Internet once a week, not getting help for depression already	Search engine advertising, Facebook ads, Forum posts, posts on relevant websites and online newsletters	14 months	Google keyword costs: Cost-per-click=AUD $0.09, cost-per-participant=AUD $10.75 (click-through rate of 6%); Google display network advertising: Cost-per-click= AUD$0.13, cost-per-person= AUD$14.71; Facebook costs: Cost-per-click=AUD $0.62, cost-per-participant=AUD $19.89 (click-through rate of 0.05%)
Ramo, Hall, & Prochaska (2010)	Comparison of three recruitment methods	201	18-25 years of age, English literate, smoked at least one cigarette in past 30 days	craigslist, Internet ads through Adbrite (2 banner ads and 1 text ad), email invitations	6 months	craigslist: Free to post (estimated $0.66 per participant for time spent); Adbrite: Cost-per-participant=$20.86 (Charge every 1000 impression); SSI (online sampling service)=$19.24 per completed survey
Ramo & Prochaska (2012)	Investigation of Facebook as a recruitment mechanism	1548	18-25 years of age, live in the United States, English literate, smoke at least one cigarette in the past 30 days	Facebook’s Ad program	13 months	Cost-per-click: $0.45; cost-per-completed survey: $4.28; Overall cost: $6,628.24
Ramo, Rodriguez, Chavez, Sommer, & Prochaska (2014)	Investigation of recruitment campaigns	79	18-25 years of age, English literate, go on Facebook 4 or more days a week, smoked 100 or more cigarettes in their lives, currently smoke one per day on 3 or more days of the week, access to camera required for bioconfirmation of nonsmoking.	Facebook’s Ads Manager program- Newsfeed ads and ads on the right column of the page	5 different standard ads, 2 sponsored stories, and 3 promoted posts were up for 3 weeks, 16 ads with a picture and text combination were posted for 7 weeks	Cost-per-click: $0.34; Overall cost: $2.024; Cost-per-participant: $8.80
Raviotta, Nowalk, Lin, Huang, & Zimmerman (2014)	Human Papillomavirus Vaccine Trial	220	18-25 years of age, male, fewer than five lifetime sexual partners, no history of HPV infection or vaccination, no autoimmune disease nor immunosuppression, no hospitalization in past year, no receipt of blood products or immunoglobulins within 90 days, no participation in other drug studies within 30 days, and no receipt of other vaccines within 8 days.	Two methods: Traditional—flyers, email, student newspaper ads, class announcements Online—Facebook ads	One 3-month period, one 5-month period	Facebook: Direct costs= $4,820, Cost-per-click= $1.24, cost-per-person= $110. Print ads and Flyers: Direct costs= $6,758, Cost-per-participant= $61
Valdez, Guterbock, Thompson, Reilly, Menefee, Bennici, Williams, & Rexrode (2014)	Feasibility trial	87	Study 1: 18 years or older, identify as Filipino, live in the United States; Study 2: 18 years or older, US citizen, diagnosed with type 2 diabetes, Facebook user	Already-established Facebook group	Study 1: 5 months; Study 2: 2 months	Study 1: No direct costs; Study 2: Direct cost= $118.17, cost-per-participant= $1.94

#### Participant Engagement and Retention

The amount of time participants spend using the site or app in a study can affect retention rates [37], and retention can greatly impact the overall power and generalizability of study results [22]. Engagement is a complex amalgam of time and interactivity with the website or app [37]. Examples of engagement metrics include number of times logging in to a website, number of page or icon clicks within an app, or completing surveys [38]. Slightly more than half (58%, 7/12) of the studies were conducting interventional research, while five (42%) of the 12 studies were conducting survey research. The studies conducting survey research (42%, 5/12) reported participant engagement as the time it took to complete study surveys. Of the remaining articles (58%, 7/12), only three (43%) reported participant engagement defined as the amount of time participants spent using the site. One intervention entailed an email chain lasting for 6 weeks [27]. Another study required a 90-day Facebook intervention and periodic biochemical confirmation of smoking cessation for 3 months [31]. The third study involved random assignment to one of two online interventions, each lasting 3 months before follow-up [29]. A third (4/12) of all the articles did not specify participant engagement at all.

The studies conducting survey research used survey completion as participant engagement. These five studies reported the number of participants that began eligibility screening, were deemed eligible, and successfully completed the surveys [25,30,32,33,36]. From these data, a completion rate was calculated as a ratio of the number of participants who completed surveys over eligible participants and then reported. These five articles (42%) either did not require or report survey follow-up, so their retention rates were calculated from their completion rates. However, retention in the context of mHealth and Web-based studies is focused on retaining participants for longer durations of time, so these rates were not reported.

Only one study (8%) reported participant retention [29]. They specified retention rates by recruitment method and found the following rates over a 3-month period: overall (52%), standard media (53%), broadcast email (46%), word of mouth (62%), press releases on health websites (56%), social media (paid ads and free posts—64%), and Google Ad Words (46%) (Tables 1 and 2). This study found no significant difference in retention rates between Web-based methods and traditional methods of recruitment [29].

**Table 2:** Summary of studies—Intervention, results, and retention.

Authors (year)	Intervention	Intervention duration	Results by recruitment method	Retention methods	Retention or completion rates
Batterham (2014)	Online survey	Time taken to complete online survey	Online surveys cost less than postal surveys- Internal links more cost less than external links. Content of ads crucial to the cost aspect of online recruitment. Terms “mental health problems” was more effective than “emotional well-being.”	Completion rate percentage calculated in order to see how many people completed surveys after responding to ads.	Survey: 10.4% completion for Problem/Altruistic ad; 11% completion for Problem/Self-Gain ad; 5.8% completion for Positive/Altruistic ad; 9% completion for Positive/Self-Gain ad
Fenner (2012)	Health survey	15-30 minutes to complete survey, either electronically or at the site	Facebook recruitment found to compare favorably with traditional recruitment methods. Facebook also found to yield a representative sample	Calculated a participation rate for those who completed the survey out of those who clicked on the ad	Survey: 3.5% participation rate out of all who clicked on ad, 65% survey completion of those who consented
Frandsen, Walters, & Ferguson (2013)	[To be reported in future publication]	[To be reported in future publication]	Most participants recruited through online methods (Facebook), Facebook cost twice as much per participant than print media. Participant demographics from each method of recruitment were equally matched meaning online methods can supplement traditional methods.	Not reported	Not reported
Graham, Bock, Cobb, Niaura, & Abrams (2006)	Telephone counseling or using QuitNet.com (an Internet smoking cessation website)	N/A	Google yielded the greatest number of participants	Completing baseline assessment	51.3% completed baseline assessment and were randomized to treatment
Graham, Milner, Saul, & Pfaff (2008)	Telephone counseling and using QuitNet.com for smoking cessation treatment	[Not reported]	Traditional methods yielded more participants and found those participants engaged with the website more, online methods reached typically hard-to-reach populations and was found to cost less	Number of logins, minutes per login, number of page views, and interactions with other users and counselors	18.4% of identifiers on QuitNet were from traditional media, 81.6% from online media. 9.1% of online clicks registered for cessation treatment (6.8% Web-only, 1.1% phone only, 1.2% both); retention data not available at time of analysis
Heffner, Wyszynski Comstock, Mercer, & Bricker (2013)	Baseline survey, 3-month follow-up assessment	Time taken to complete baseline survey and follow-up 3 months later	GoogleAds had highest participant yield, social media and GoogleAds cost more than traditional methods. No difference between traditional and online methods in data retention or intervention success	Completing follow-up after 3 months went by	3-month retention: Overall: 52%, Standard media: 53%, email: 46%, word of mouth: 62%, medical Internet: 56%, social media: 64%, Google: 46%
Morgan, Jorm, & Mackinson (2013)	Patient Health Questionnaire, receiving weekly emails with self-help strategies	6 weeks	Google had highest participant yield, found to be less time-consuming and more effective than other recruitment techniques, even those that are free.	Completing baseline survey, receiving emails, and completing depression questionnaire	Survey: 78% completion after consent
Ramo, Hall, & Prochaska (2010)	10-item smoking questionnaire; Fagerstron Test of Nicotine Dependence; smoking stages of change questionnaire	20 min survey	Adbrite Internet advertisements resulted in highest participant yield (Facebook was most successful Internet Web sites), craigslist and SSI were more successful at targeting young adult smokers	Completing survey in entirety (all questionnaires)	Survey: 59.8% completion after eligibility screening
Ramo & Prochaska (2012)	Smoking Stages of Change Questionnaire, A Smoking Questionnaire, Thoughts about Abstinence form	Time to complete survey	Facebook found to be more affordable than other previously- used methods with a similar population (Internet marketing company cost-per-participant=$42.77), success of specific ads dependent upon Facebook’s approval of the ad	Completing survey in entirety	Survey: 50% completion after eligibility screening
Ramo, Rodriguez, Chavez, Sommer, & Prochaska (2014)	Smoking History Questionnaire, Smoking States of Change scale, baseline survey, Private Facebook group tailored to their readiness to quit (ready, thinking about it, not ready), bioconfirmation of nonsmoking tests	One year	Facebook found to be efficient and affordable. Newsfeed ads more successful—Ads with simple pictures of cigarettes, the study logo, and general info messaging were most successful	Participate in a Facebook group and complete follow-up assessments with saliva cotinine tests at 3, 6, and 12 months.	34% of those who were eligible and consented completed the intervention
Raviotta, Nowalk, Lin, Huang, & Zimmerman (2014)	Complete baseline survey and postvaccination survey. Half participants randomized to standard dosing group (0, 2, & 6 months) and half randomized to alternate dosing group (0, 2, & 12 months).	[Not reported]	Traditional methods reached more people, but online methods were more likely to reach hard-to-reach populations. Direct cost was higher for electronic advertising	Completing four visits to study site for intake survey, blood draw, vaccination does 1, 2, & 3, and final blood draw and survey	Survey: 70.7% completion after eligibility screening
Valdez, Guterbock, Thompson, Reilly, Menefee, Bennici, Williams, & Rexrode (2014)	Survey	Time taken to complete the survey	Facebook found to be affordable, but not feasible for large, quantitative studies	Completing the survey in its entirety	Survey: 77.2% completion after eligibility screening

## Discussion

### Principal Findings

The use of mHealth has tremendous potential for improving public health through its convenience, wide reach, and flexibility [14]. The Internet and mHealth apps are increasingly used by individuals who seek health information, thus increasing the potential to reach underserved populations [32]. However, there is a need to develop and evaluate mHealth apps in order to establish consistent, effective methods for producing health behavior change. Research on mHealth may experience challenges in participant recruitment and retention due to its very nature, which is characterized by the virtual aspect of the intervention [27]. Virtual interventions may lead to less investment and fraudulent enrollment on behalf of participants due to lack of relationship with the study team and potential incentives to participate [27]. Personal relationships may help participants to understand their contribution to the research [27]. Currently the literature on Web-based interventions is greater than that for mHealth apps, but there is a lack of detail in both about participant recruitment and even less information on retention. More attention must be paid to these two factors as they affect the validity and generalizability of the research findings [15].

### Cost of Recruitment

For researchers conducting online and/or mHealth studies, online methods of recruitment have the potential to achieve better affordability than traditional methods of recruitment [27]. However, the results of this review found inconsistent findings related to cost-per-participant. This review also found conflicting outcomes regarding whether Web-based or traditional methods of recruitment were more effective at enrolling participants. It appears that the type of intervention and target population influenced which type of recruitment method was most successful.

Of the 12 studies examined, there was no clear consensus on which method (Web or traditional) is best to use when conducting mHealth research. The success of recruitment method varied widely based on population, budget, intervention, cost, and study design. “Best method”, as defined in these articles, was the one that achieved the highest participant yield for the cost incurred. Assessing largest participant yield can be achieved through analyzing overall impressions and click-through rates. Four articles (33%) found Facebook ads to be effective for recruitment when the intention was to achieve a sample within a specific age range. The use of Facebook ads for recruiting those under 25 may be due to the fact that Facebook offers the ability to target a specific demographic or age group by displaying ads only on profiles within that listed age range. Google ads appeared to be the most effective Web-based recruitment method when there was no specific age range targeted. Four studies (33%) compared Web-based methods against traditional methods of recruitment and reached different conclusions about highest participant yield and affordability.

There are a variety of factors that could lead to these results. First, paying for advertisements is different for each online method. For example, the bidding method that both Facebook and Google use does not allow for a stable cost prediction that could be replicated by the same researcher or even future researchers. This is due to the intermittent presence of other bidders. Based on project budgets and desired participant demographics, very different results could be achieved. In addition, the business models of Internet advertising (eg, Facebook and Google ads) are constantly changing, presenting ongoing challenges for determining affordable strategies relating to online recruitment. Also, virtual interventions and methods of recruitment allow for a certain degree of ambiguity and anonymity. This might allow participants to feel more comfortable enrolling in a study or could lead to less investment in the process.

### Participant Retention

Overall, studies are not doing a good job of reporting retention rates. Only one of the five studies that utilized a long-duration intervention reported participant retention rates. Retention issues can create bias and complications with generalizability [22], and if the rates are not reported, it is unclear if the results are valid. Consort diagrams are needed to describe the recruitment process and sources of attrition. Retention is a factor that not only should be addressed, but also reported with its potential effects in all articles related to online (eHealth) and mHealth interventions.

We recommend that researchers report recruitment methods, costs, participant demographics, and participant engagement metrics in their publications. Studies evaluating the effectiveness of interventions, especially mHealth interventions, should include this information in order to allow for future replication. A set of guidelines should be established for informing eHealth and mHealth researchers about recruiting generalizable samples. These guidelines might include, for example, a list of successful methods for targeting a specific participant demographic or approaches to maximizing a limited recruitment budget.

### Limitations

This review has two possible limitations. First, the limited number of studies required us to modify the selection process to more broadly examine the literature in order to meet the specific needs of the research team. Therefore, our inclusionary and exclusionary criteria changed over the course of the study. Second, the studies identified during the search used very different designs and methods. These discrepancies made it difficult to form recommendations as to which methods of recruitment are most beneficial and cost-effective in conducting mHealth research.

### Future Research and Recommendations

Further evaluation of online methods of recruitment is necessary to better understand their effectiveness for use in Web-based and mHealth research. Due to the unique challenges that Web-based and mHealth interventions face, there is a critical need for published articles focusing solely on recruitment methods, including participant recruitment and retention rates. Although it was outside the scope of this review, researchers in this area may also experience the possibility of fraudulent participants. Little is known about how the mHealth research process may encourage/discourage “fake” participants from enrolling in this type of study. There is also a need for guidelines and recommendations for affordable ways of recruiting and retaining representative samples of participants in Web-based and mHealth research. It is difficult to assess which methods of recruitment will work best, and there is not a “one-size-fits-all” approach, which should be included as a part of a set of guidelines. Currently, recommendations for guidelines cannot be made due to the inconsistent nature of reporting of online recruitment strategies. As the body of literature grows around mHealth, and as researchers begin to report specific recruitment methods, associated costs, and retention methods and rates, guidelines may be formed.

### Conclusions

Research on mHealth apps and Web-based interventions may experience challenges with participant recruitment and retention. The research available regarding ideal participant yield and the associated costs of online recruitment methods for Web-based and mHealth studies is minimal and inconsistent. Researchers in this area should routinely report metrics regarding recruitment methods used and participant attrition. This includes detailing specifically what type of ads were used (eg, banner or search ads), as well as specific cost information (eg, cost per click, cost per participant, and overall costs). In addition, retention methods and retention rates should be included, as it is an important factor for mHealth and Internet-based studies. Information needed would include participant engagement in the intervention (eg, metrics associated with use of the intervention), and how many participants successfully completed the intervention and associated research activities. A set of guidelines for successful and affordable methods of recruitment, and metrics for evaluating mHealth engagement and participant retention are needed.
